# Independent and interactive associations of dietary nitrate and salt intake with blood pressure and cognitive function: a cross-sectional analysis in the InCHIANTI study

**DOI:** 10.1080/09637486.2021.1993157

**Published:** 2021-11-16

**Authors:** Andrea M. McGrattan, Blossom C. M. Stephan, Oliver M. Shannon, Mohsen Mazidi, Mark Gilchrist, Miranda Smallwood, Paul Winyard, Nicholas McMahon, Lauren C. Blekkenhorst, Devi Mohan, Stefania Bandinelli, Louise Robinson, Luigi Ferrucci, Mario Siervo

**Affiliations:** a School of Biomedical, Nutritional and Sport Sciences, Faculty of Medical Sciences, Newcastle University, Newcastle upon Tyne, UK; b School of Medicine, University of Nottingham, Nottingham, UK; c Faculty of Medical Sciences, Human Nutrition Research Centre, Population Health Sciences Institute, Newcastle University, Newcastle upon Tyne, UK; d Department of Twin Research & Genetic Epidemiology, King’s College London, London, UK; e Nuffield Department of Population Health, Clinical Trial Service Unit and Epidemiological Studies Unit (CTSU), University of Oxford, Oxford, UK; f Institute of Biomedical and Clinical Sciences, University of Exeter and NIHR Exeter Clinical Research Facility, Exeter, Devon, UK; g College of Medicine and Health, University of Exeter, Exeter, UK; h School of Human Movement and Nutrition Sciences, University of Queensland, St. Lucia, QLD, Australia; i School of Medical and Health Sciences, Institute for Nutrition Research, Edith Cowan University, Perth, WA, Australia; j Medical School, The University of Western Australia, Perth, WA, Australia; k Global Public Health, Jeffrey Cheah School of Medicine and Health Sciences, Monash University Malaysia, Bandar Sunway, Malaysia; l Laboratory of Clinical Epidemiology, InCHIANTI Study Group, LHTC Local Health Tuscany Center, Florence, Italy; m Faculty of Medical Sciences, Population Health Sciences Institute, Newcastle University, Newcastle up-on Tyne, UK; n Biomedical Research Centre, National Institute on Aging, NIH, Baltimore, MD, USA; o School of Life Sciences, University of Nottingham, Nottingham, UK

**Keywords:** Dietary nitrate, salt, sodium, cognition, ageing, blood pressure, biomarkers

## Abstract

Blood pressure (BP) control is a key target for interventions to reduce cognitive decline. This cross-sectional study explored associations between objective (24-hour urine excretion) and subjective (food frequency questionnaire [FFQ]) measures of dietary sodium and nitrate intakes with cognitive function and resting BP in the InCHIANTI cohort. Baseline data from 989 participants aged >50 years were included. In fully adjusted models, participants with concurrent high nitrate and low sodium (Odds Ratio (OR)=0.49, 95%CI 0.32–0.76, *p* = 0.001) and high nitrate and high sodium (OR = 0.49, 95%CI 0.32–0.77, *p* = 0.002) 24-hour urinary concentrations had lower odds of high BP than participants with low nitrate and high sodium concentrations. We found no significant associations between sodium and nitrate intakes (24-hour urinary concentrations and FFQ) and poor cognitive performance. Urinary nitrate excretion was associated with lower BP and results appeared to be independent of sodium intake. Further analyses in longitudinal studies are required to substantiate these findings.

## Introduction

Blood pressure (BP) control has emerged as a key target for long-term, effective interventions to reduce cognitive decline and prevent dementia (Hughes et al. 2020). Hypertension in mid-life has been associated with increased dementia risk (Livingston et al. 2020), with a recent clinical trial reporting a lower risk of mild cognitive impairment among individuals randomised to an intensive BP reduction intervention (Williamson et al. 2019). However, data on the association of BP control with cognitive decline are inconsistent in older individuals.

Dietary sodium (salt) and inorganic nitrate both influence BP control (Strazzullo et al. 2009; Lundberg et al. 2011). Epidemiological studies have reported associations of high sodium diets with impaired BP control and increased cardiovascular risk (Strazzullo et al. 2009; Mente et al. 2018); this evidence has been confirmed in several clinical trials demonstrating the protective effects of salt reduction on cardiovascular health (He et al. 2013; He and MacGregor 2018). Dietary nitrate is an exogenous source for the endogenous production of a substrate for, and metabolite of, nitric oxide (NO), which is known for its multiple effects on physiological functions such BP, neurovascular coupling, neurotransmission and immunity (Clifford et al. 2019). Recent epidemiological studies and clinical trials have demonstrated nitrate intake improves BP and metabolic health (Babateen et al. 2018; Clifford et al. 2019).

While the scientific evidence for the benefits of dietary sodium reduction and increased dietary nitrate intake on BP appears robust, relatively little is known about the association of both sodium and nitrate intake with cognitive decline. To date, a small number of epidemiological studies among older adults have been conducted and showed mixed associations between sodium intake and cognitive performance (Fiocco et al. 2012; Haring et al. 2016; Rush et al. 2017; Nowak et al. 2018). The association of dietary nitrate with cognition is largely unknown. A small number of clinical interventions have been conducted reporting mixed results however, they had overall a short duration and limited statistical power (Stanaway et al. 2017; Clifford et al. 2019).

Nevertheless, with hypertension highlighted as an important modifiable risk factor for dementia (Livingston et al. 2020) and the consistent beneficial effects of low sodium and high dietary nitrate intakes on BP, it is relevant to investigate their independent and combined putative associations with cognitive outcomes. The consumption of a diet low in sodium and high in nitrate could potentially represent a more effective dietary strategy to achieve greater reductions in BP and provide greater benefits in cognitive function. To our knowledge, the interactive effects between dietary nitrate and sodium intake are yet to be explored in humans.

In addition, the mixed results observed to date on the associations of dietary nitrate and sodium intake with health outcomes may be due to differences in dietary assessment methods across studies. In particular, results could differ depending upon whether subjective, self-reported data measures of dietary intake are utilised (e.g. food frequency questionnaire (FFQ) or diet recall) compared with objective measures such as 24-hour urinary sodium or nitrate concentrations (Hill et al. 1996; Wielgosz et al. 2016). Inaccuracies, inconsistencies, limited and/or poor-quality food composition tables to calculate dietary nitrate intake may result in misreporting of nitrate intake. To our knowledge, a cross-validation analysis to evaluate associations between FFQ and 24-hour urinary measurements of sodium and nitrate intake has not been conducted.

The aim of this cross-sectional study was to explore associations between objective (i.e. 24-hour urine excretion) and subjective (i.e. self-reported FFQ) measures of dietary sodium and nitrate intakes with cognitive function and BP among older adults (>50 years) from the InCHIANTI cohort. In addition, this study aimed to conduct a cross-validation to evaluate the comparability of measures of nitrate and sodium intake derived from FFQ vs. 24-hour urine concentrations.

## Methods

### Study population and setting

The InCHIANTI study is a population-based epidemiological investigation among an older adults living in the Chianti region in Tuscany, Italy. The details of the study have been previously reported [18]. The study was conducted by the Laboratory of Clinical Epidemiology of the Italian National Institute of Research and Care on Ageing (INRCA), Florence, Italy. Ethical approval was granted by The INRCA Ethical Committee. The InCHIANTI study aimed to recruit older residents from two towns of the Chianti area (Greve in Chianti and Bagno a Ripoli, Tuscany, Italy) plus younger controls, and achieved a 91.6% response rate at baseline (data collected between 1998 and 2000). 1453 individuals from 20 to 102 years of age were randomly selected based on city registries [18].

### Participant selection

**Figure 1.**
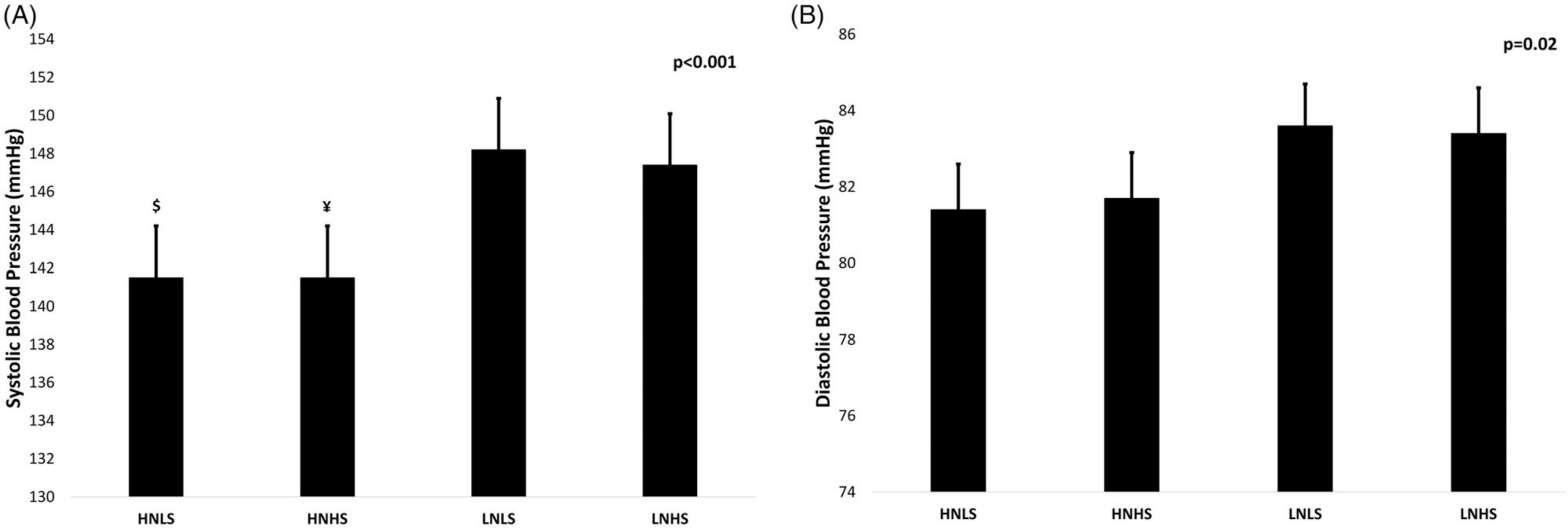
Interactive effects of differences in 24-hour urinary concentrations of nitrate and sodium on mean systolic (A) and diastolic (B) resting blood pressure. Analysis of covariance was conducted to evaluate differences between the groups. Analyses were adjusted for age, sex, disease count score (stroke, CHF, MI, PD, cancer and diabetes), BMI, physical activity, total energy intake, smoking, kidney function and medication use (drugs for acid-related disorders; diuretics for CHF or hypertension; ACE inhibitors (alone and in combination with diuretic); organic nitrates; aldosterone antagonists (diuretics); glucocorticoids for systemic use). $=Significant difference from LNLS and LNHS (*p* < 0.05). ¥ = Significant difference from LNLS and LNHS (*p* < 0.05). *Key*: HNLS: high nitrate low sodium; HNHS: high nitrate high sodium; LNLS: low nitrate low sodium; LNHS: low nitrate high sodium.

For this cross-sectional analysis, we used data from *n* = 1270 participants older than 50 years at the baseline interview. In addition to age, the following exclusion criteria were applied: (1) total urine volume <400 or >6000 ml; (2) total energy intake >800 and <4000 kcal/day (Fiocco et al. 2012); (3) poor renal function (eGFR < 30 ml/min). Following application of exclusion criteria, the number of participants with complete cognitive assessment data (Mini Mental State Examination [MMSE]; Trail Making Task [TMT A and TMT B] respectively) and BP measurements at baseline varied within the dataset. Flowcharts of study participants eligible for inclusion for each outcome are illustrated in Figures 1(A–D) of the online supplementary material.

### Blood pressure

Resting supine BP was measured in both arms using a mercury sphygmomanometer. The sequence of measurements was right arm first, then a 2-minute pause, then the left arm. Two further measurements were subsequently carried out on the higher reading arm. Systolic and diastolic BP were defined as the mean of the second and third measurements. All BP measurements were taken from the initial baseline recruitment examinations.

### Cognitive function

Cognition was assessed at baseline using two measures: (1) an Italian version of the Mini Mental State Examination (MMSE) (Folstein et al. 1975). MMSE assesses global cognition including orientation, registration, attention, calculation, language and recall. MMSE scores range from 0–30 with higher scores indicating better cognitive function; (2) Trail Making Test (TMT) (Reitan 1958). TMT was administered to measure visuospatial scanning, sequential processing, motor speed, attention, executive functioning and is administered in two parts (TMT A and TMT B). In TMT A, the participant was asked to draw lines to connect circled numbers in a numerical sequence (i.e. 1–2-3, etc.), and in TMT B, participants were asked to draw lines to connect numbers and letters in alternating order. TMT A and B was completed as quickly as possible, and scores reflected the time needed to complete the task with 300 seconds as the maximum. Higher scores indicated poorer performance. The difference between TMT A and TMT B scores was calculated to account for missing data in the completion of the TMT B test.

### Dietary assessment measures

#### 24-Hour urinary sodium

Sodium intake was estimated using the 24-hour sodium urinary excretion. On the day of the study visit, participants were provided with a plastic bottle containing 1 g of boric acid as preservative, and instructed to collect all the urine produced in the following 24 hours, making the maximum effort to avoid dispersing urine during the collection period.

#### 24-Hour urinary nitrate

Urinary nitrate concentration was estimated by the 24-hour nitrate urinary excretion and measured using the spectrophotometric plate method which has been described elsewhere (Smallwood et al. 2017). Briefly, the assay reduces the nitrate in the sample to nitrite which then forms a coloured chromogen upon reaction with the Griess reagent. Absorbance was read using a plate reader at 540 nm. Thus, the assay does not differentiate between nitrite and nitrate. As the concentration of nitrate is approximately 1,000 times that of nitrite in urine a ratio that at the very least persists following nitrate supplementation we have reported the results as urinary nitrate concentration (Smallwood et al. 2017).

#### Dietary intake from FFQ. 

Usual dietary consumption in the past year was assessed at baseline using a FFQ created for the European Prospective Investigation on Cancer and Nutrition study and validated for the InCHIANTI study (Pisani et al. 1997; Bartali et al. 2004). Daily sodium intake was calculated in mg/day. Nitrate data is not included within national food composition tables; therefore a published comprehensive database that includes the nitrate and nitrite concentrations in 3498 and 2134 individual food and beverages, respectively, was used to calculate dietary nitrate intake (mg/day) (McMahon et al. 2017).

#### Covariates. 

Factors that were associated with cognitive function and BP in univariate analyses or known to be associated from previous studies were considered as covariates in the analyses (Tanaka et al. 2018; Clark et al. 2020). Sociodemographic covariates included age (years), sex (male or female), and number of years of education. Height (m) and weight (kg) were measured and used to calculate Body Mass Index (BMI) (kg/m^2^). Total energy intake was assessed using FFQ data (kcal/day). Lifestyle factors included physical activity levels in the 12 months prior to participants’ baseline visit, assessed through a modified standard interview-administered questionnaire and coded into three categories of low activity (inactivity or light-intensity activity <1 h per week), medium activity (light-intensity activity 2–4 h per week), and high physical activity (light-intensity activity at least 5 h per week or moderate activity at least 1–2 h per week) (Wareham et al. 2002); smoking status was self-reported and categorised into never smokers, former smokers or current smokers (smoking within 3 years of interview); depressive symptoms were measured using Centre for Epidemiological Studies Depression (CESD) scores (0–60; higher scores related to greater depressive symptoms). Use of medications (yes/no) that may alter metabolism of sodium or nitrate in the body were controlled for, including drugs for acid-related disorders; diuretics for Congestive Heart Failure (CHF) or hypertension; ACE inhibitors (alone and in combination with diuretic); organic nitrates; aldosterone antagonists (diuretics); glucocorticoids for systemic use; antiepileptic; anti-Parkinson drugs; psycho-leptics: typical antipsychotics; psycho-leptics: atypical antipsychotics; psycho-leptics: anxiolytics; psycho-analeptics: antidepressants; drugs for dementia. Chronic diseases including, diabetes, ischaemic heart disease, congestive heart failure, stroke, cancer, Parkinson’s disease, were defined using standard clinical definitions which have been described previously (Fabbri et al. 2015). For the analysis, a cumulative count of the conditions present in each participant was calculated as an indicator of general health status. Estimated Glomerular Filtration Rate (eGFR) was estimated using the Cockcroft–Gault equation and used to classify kidney function (Foundation 2002).

#### Data analysis.

**Figure 2.**
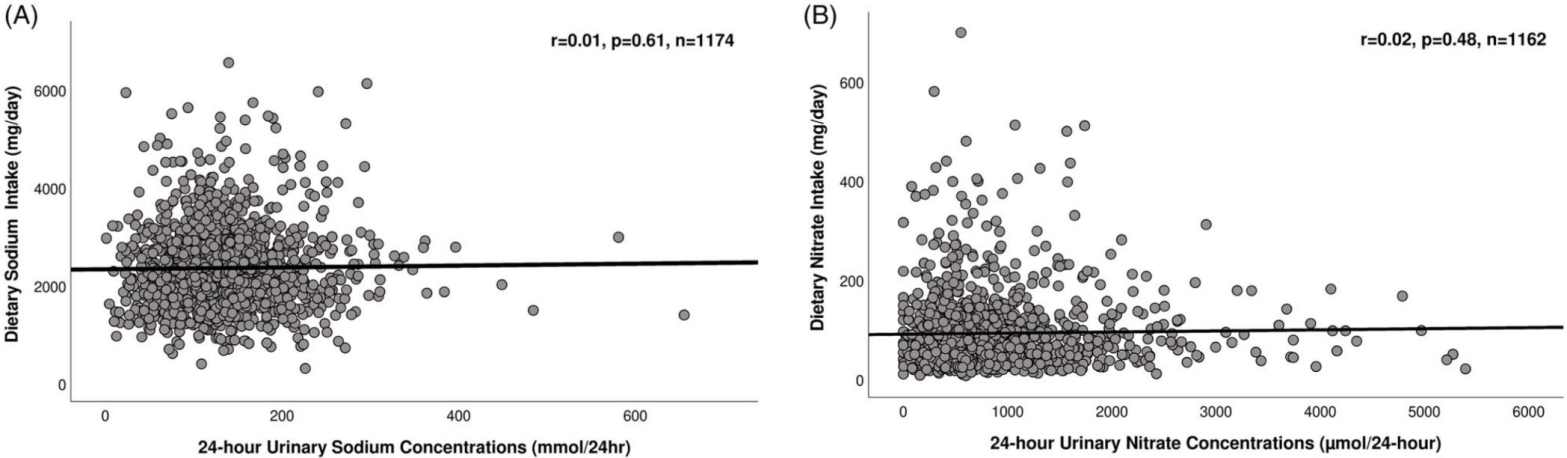
Scatter-plots showing the associations between 24-urinary sodium and nitrate concentrations with dietary sodium (A) and nitrate (B) intake assessed by Food Frequency Questionnaire.

Statistical analyses were conducted using IBM SPSS version 25.0 (New York, NY, USA). The threshold for significance was set at *p* ≤ 0.05. Descriptive statistics were summarised as mean and SD or frequency and percentages. Histograms of the distributions of 24-hour urinary sodium and nitrate excretions and dietary sodium and nitrate intakes are presented in Figure 2(A–D) of the online supplementary material. Scatter plots were produced to evaluate the associations of measurements of nitrate and sodium intake with 24-urinary excretion of nitrate and sodium, respectively.

Multiple linear regression was used to investigate the association between urinary sodium, urinary nitrate, dietary sodium, and dietary nitrate with cognitive test scores and BP. Results are presented as estimate and standard error. Models were reported as adjusted for age, sex, disease count, medication use, BMI, physical activity, energy intake, smoking, education, depression, kidney function and BP (for cognition analysis only). Normality of the residuals distributions was checked to evaluate the fitness of the regression models.

Logistic regression was used to calculate the odds ratio (OR) for the association between urinary sodium, urinary nitrate, dietary sodium and dietary nitrate with poor cognitive performance and high BP. Urinary nitrate, dietary nitrate, urinary sodium and dietary sodium were stratified into tertiles based on their frequency of distribution in the data set. Poor MMSE cognitive performance was defined as a score in the bottom 20^th^ percentile of the population distribution for MMSE (Gross et al. 2019). According to the time (in seconds) employed to perform each part of the TMT, poor performance was defined as a score in the 20^th^ percentile of the population distribution as higher scores demonstrate poorer performance (more time taken to complete). BP was categorised according to the European Society of Cardiology Guidelines (Williams et al. 2018), with measurements for Systolic BP (SBP) of ≥140 mmHg and/or Diastolic BP (DBP) of ≥90 mmHg classed as high BP. Results are expressed as ORs with 95% confidence intervals (CIs) for poor cognitive performance/high BP with tertiles 2 and 3 compared with tertile 1 (lowest) of urinary or dietary nitrate/sodium. Results from unadjusted and fully adjusted models were reported. Covariates included in the models were age, sex, disease count score, medication use, BMI, physical activity, energy intake, smoking, education, depression, kidney function and BP (for cognition analysis only). Normality of the residuals distributions was checked to evaluate the fitness of the regression models.

The interaction effect of intakes of sodium and nitrate were assessed via logistic regression. Four intake groups we created based on median values of sodium (135 mmol/24 hr) and nitrate (667 mmol/24 hr) urinary excretion (High intake = above median value; Low intake = below median value), and reclassified into the following: Group 1, High Nitrate and Low Sodium (HNLS); Group 2, High Nitrate and High Sodium (HNHS); Group 3, Low Nitrate and Low Sodium (LNLS) and Group 4, Low Nitrate and High Sodium (LNHS) (reference group). The same analysis process was followed as described previously to calculate the OR for the intake groups and poor cognitive performance or high BP. Finally, ANCOVA analyses were used to determine mean differences between the intake groups and, if significant, *post-hoc* (Bonferroni) tests were conducted to identify differences between dietary groups. ANCOVA analyses were adjusted for age, sex, disease count score, medication use, BMI, physical activity, energy intake, smoking, education, depression, kidney function and BP (for cognition analysis only).

## Results

### Participant characteristics

Table 1 shows the demographic and health characteristics of the InCHIANTI participants included in this analysis. The mean age of the sample was 73.5 years, with a higher percentage being female (54.3%), married (63.2%) and non-smokers (57.4%). The majority reported elementary school as the highest level of education completed (51.9%), followed by 27.6% who reported no schooling, with a mean of 5.6 years of school completed across the sample. On average, participants were overweight (27.2 ± 4.1 kg/m^2^) and had a total daily energy intake of 1996 ± 602 kcal/day. The majority were moderately physically active (41.5%) and had relatively low CESD depression scores (12.8 ± 8.9). Most participants (61.2%) had no comorbidities (including myocardial infarction, coronary heart failure, stroke, cancer, Parkinson’s disease, or diabetes); 36.9% reported between 1 and 2 comorbidities. On average, SBP was 145 ± 21 mmHg, DBP was 82 ± 9 mmHg and eGFR was 67 ± 20 mL/min across the sample. For cognitive function, on average participants scored 24.5 ± 5.2 on MMSE, 29.9 ± 16.5 seconds on TMT A (*n* = 889) and 28.9 ± 18.2 seconds on TMT B (*n* = 721). Urinary excretions of dietary nitrate and sodium averaged at 862 ± 734 mmol/24 hr (median: 670.4 mmol/24 hr, IQR: 650.2 mmol/24 hr) and 139.5 ± 65.7 mmol/24 hr (median 134.5 mmol/24 hr, IQR: 75.2 mmol/24 hr), respectively. Intakes of dietary nitrate and sodium as estimated by FFQ averaged at 89.0 ± 73.3 mg/day (median: 67.5 mg/day, IQR: 67.9 mg/day) and 2358 ± 849 mg/day (median: 2266.4 mg/day, IQR: 1014.2 mg/day), respectively.

### Associations between sodium and nitrate intake and cognitive function

Urinary sodium and dietary sodium intake were not associated with any cognitive performance test scores (MMSE, TMT A or TMT B) in unadjusted and fully adjusted linear regression models. Similarly, for urinary nitrate and dietary nitrate, there were no associations with any cognitive function test scores (Table 2).

### Associations between sodium and nitrate intake and BP

Urinary sodium and dietary sodium intake were not associated with BP (SBP or DBP). Urinary nitrate was inversely associated with SBP (−0.005 ± 0.001 mmHg, *p* < 0.001) and DBP (−0.001 ± 0.001 mmHg, *p* = 0.002) in fully adjusted models. No significant associations were observed for dietary nitrate and BP (Table 3).

### Associations between sodium and nitrate intake and impaired cognitive function

No significant associations were observed for tertiles of urinary sodium and nitrate concentrations and for dietary sodium and nitrate intake with odds of cognitive impairment (i.e. MMSE, TMT-A, TMT-B and TMT A-B) (Table 1 of the online supplementary material).

### Associations between sodium and nitrate intake and high BP

Urinary sodium concentrations and dietary sodium and nitrate intake were not associated with BP. For 24-hour urinary nitrate concentrations, those in tertile 2 (OR = 0.57, 95%CI 0.37–0.88, *p* = 0.01) and tertile 3 (OR = 0.31, 95%CI 0.21–0.48, *p* < 0.001) had a lower odds of high BP in comparison to those in tertile 1 (Table 4).

### ANCOVA analysis: differences in cognitive function and BP between groups

There were no statistically significant differences between groups for cognitive performance (all tasks) (Table 5 of the online supplementary material). Statistically significant differences between groups for both SBP (*p* < 0.001, Figure 1(A)) and DBP (*p* = 0.041, Figure 1(B)) were found in fully-adjusted models (Table 5 of the online supplementary material). The HNLS had significantly lower mean SBP compared to LNLS (−6.7 mmHg, 95%CI −12.4, −1.0 mmHg; *p* = 0.003) and LNHS (−6.1 mmHg, 95%CI −11.8, −0.4 mmHg; *p* = 0.018) groups. Similarly, the HNHS group showed lower mean SBP compared to LNLS (−6.7 mmHg, 95%CI −11.8, −1.6 mmHg; *p* = 0.003) and LNHS (−5.9 mmHg, 95%CI −11.1, −0.6 mmHg; *p* = 0.017) groups (Figure 1(A)).

### Association of dietary nitrate and sodium intake with 24-hour urinary nitrate and sodium concentrations

Dietary nitrate and sodium intake assessed by FFQ were not associated with 24-hour urinary sodium (*r* = 0.01, *p* = 0.61, Figure 2(A)) and nitrate (*r* = 0.02, *p* = 0.48, Figure 2(B)) concentrations, respectively.

## Discussion

In this cross-sectional analysis, sodium and nitrate intake, derived from FFQ data and measurement of 24-hour urinary concentrations, was not associated with cognitive performance in the InCHIANTI study population. In addition, no significant association was found with BP for dietary nitrate intake measured by FFQ and both measures of sodium intake (24-hour urine and FFQ). However, higher concentrations of 24-hour urinary nitrate were associated with lower systolic and diastolic BP values, indicating that objective biomarkers of nitrate intake may be more sensitive measures of dietary exposure to detect significant associations with health outcomes. This study also explored the interactive effects between sodium and nitrate 24-hour urinary nitrate concentrations on BP and cognitive performance. While no significant dietary interactions were found with cognitive performance, there was a significant association of the high nitrate groups (HNLS and HNHS), independent of sodium intake, on mean systolic and diastolic BP and a lower odds of high BP in both groups with higher 24-hour urinary nitrate concentrations.

Recent observational studies have explored the association between sodium intake and cognitive performance and cardiovascular outcomes to find mixed results (He and MacGregor 2018; Kendig and Morris 2019; Mohan et al. 2020). A study by Lelli et al, (Lelli et al. 2018) conducted in the InCHIANTI population (*n* = 920) found no association between 24-hour urinary sodium excretion and 9-year incidence of cardiovascular diseases (adjusted risk ratio 0.96, 95% CI 0.90–1.02). Conversely, a smaller study (*n* = 119) reported a significant association between higher urinary sodium excretions with lower MMSE scores (Afsar 2013). Other studies have evaluated associations between cognitive performance and sodium intake using data derived from FFQs. For example, a longitudinal study among community dwelling older adults (Rush et al. 2017) reported a significant association between lower dietary sodium intake with poorer performance on tests of global (MMSE) and executive (Trails B) cognitive function after controlling for age, sex, and education. The NuAGE study (Fiocco et al. 2012) investigated the association of physical activity, sodium intake and cognitive function and found that a higher sodium intake was associated with a greater 3-year decline in global cognitive function, but only in individuals with low physical activity levels. Nowak et al., (Nowak et al. 2018) evaluated the associations of dietary sodium, potassium and ratio of sodium: potassium intake with cognitive decline among community-dwelling older adults from the Health, Ageing and Body Composition (ABC) study. The study showed that higher sodium: potassium intake, but not sodium or potassium intake alone, was associated with decline in cognitive function, with no associations observed with micro- and macro-structural brain MRI indices. Finally, a prospective follow up study among 6,426 cognitively normal older women from the Women’s Health Initiative Memory Study (WHIMS) (Haring et al. 2016) demonstrated that sodium intake did not modify the risk for cognitive decline in women with hypertension or receiving antihypertensive medication.

Recent observational studies have explored the association of urinary nitrate with cardiovascular outcomes and cognitive performance. Smallwood et al., (Smallwood et al. 2017) showed in the InCHIANTI study a lower diastolic (−1.9 mm Hg) and systolic (−3.4 mm Hg) BP in participants with urinary nitrate excretion greater than 2 mmol. In addition, two independent analyses conducted in the NHANES dataset found that urinary nitrate concentrations in spot samples were associated with a lower prevalence of hypertension, stroke and congestive heart failure (Mendy 2018; Wu et al. 2020). These findings are consistent with the results presented here, with significant associations of 24-hour urinary nitrate with systolic BP but this was not seen in analyses with self-reported dietary nitrate derived from the FFQ.

A cross-sectional study among the NHANES cohort (Pereira et al. 2021) found that urinary nitrate concentrations were not associated with cognitive performance which is consistent with the results reported in this paper.

The interaction between dietary sodium and nitrate 24-hour urinary concentrations produced interesting but also unexpected results. Both high-nitrate groups were associated with lower BP values and results were not modified by sodium concentrations. We also tested whether significant interactive associations were observed using dietary sodium and nitrate intake data from the FFQs but results were not significant (Supplementary Material).

These findings certainly merit a discussion on the sensitivity of the dietary assessment methods for both sodium and nitrate. We have showed in this population a lack of association between FFQ measures of intake for both nitrate and sodium and 24-urinary concentrations. The association of 24-hour urinary nitrate concentrations with lower BP readings are aligned with results found in previous studies, which seems to indicate a greater sensitivity of objective biomarkers of nitrate intake to ascertain associations with relevant health outcomes. The lack of a significant association of the FFQ method could be partly due to its ability to capture long-term exposure to dietary factors, which may not be ideal in cross-sectional studies to evaluate associations with health outcomes, especially when both exposures and outcomes may be characterised by short biological half-lives and prone to significant variability between measurements (Satija et al. 2015; Bennett et al. 2017). Furthermore, sodium intake could be underestimated by FFQ assessments, as it may not accurately capture when salt is added to meals, whereas nitrate intake could be overestimated as individuals tend to misjudge their intake of vegetables when questioned (Cobb et al. 2014). Measurements of dietary exposure in 24-hour urinary samples, if collected alongside the measurements of the outcome variables, may provide a more accurate measure of dietary exposure to test associations with health outcomes. Repeated 24-hour dietary recalls may provide a superior proxy of dietary intake if collected alongside outcome measurements and collection of biological samples. However, FFQ may represent a more sensitive method to investigate associations in longitudinal analyses as they might be better proxy of a life-long, typical dietary exposure in an individual and hence more suitable to investigate associations over prolonged follow up periods. These important methodological questions require further investigation in carefully designed validation studies.

A discussion of the biological mechanisms that may explain the greater association of dietary nitrate with BP outcomes compared to sodium intake is relevant. Both sodium and nitrate influence NO production and affect endothelial integrity and vascular tone (Oberleithner et al. 2007; DeMartino et al. 2019). Their respective roles are however contrasting as an increase in nitrate intake determines, via a two-step reduction process, the formation of nitrite first (via facultative anaerobic bacteria on the dorsal surface of the tongue) and then conversion into NO (stomach or peripheral circulation), which increases the dephosphorylation of myosin lighter chains and Ca^2+^ reuptake into the sarcoplasmic reticulum leading to vaso-relaxation (Chen et al. 2008). On the other hand, the association of sodium intake with BP seems to have a J-shaped association as both low and high sodium intakes may have negative effects on mediators of vascular resistance and fluid homeostasis (i.e. autonomic regulation, renin-angiotensinin-aldosterone system, oxidative stress, extracellular matrix remodelling), which may explain the contrasting associations between dietary sodium intake and cardiovascular outcomes found in the literature (Edwards and Farquhar 2015). While the role of low sodium intake was not explored in this analysis, it is still unexpected the lack of association between high sodium intake and BP and the sodium-independent, protective roles of high dietary nitrate intake in this population. It is conceivable that the biological effects of the high-sodium diets may have been influenced by several factors affecting sodium-handling in individuals, including salt-sensitivity, genetic polymorphisms and nutrient-nutrient interactions (i.e. potassium or magnesium) (Morrison and Ness 2011). The physiological mechanisms involved in the control of vascular homeostasis during the co-ingestion of nitrate (normal and high intakes) and sodium (low, normal and high) need investigation in carefully controlled feeding studies.

### Strengths and limitations

This is the first study to investigate the interactive effects of sodium and nitrate intake, using both biomarkers and FFQ derived measures of intake, with cognitive performance and BP. The inclusion of a large population-based sample with validated and standardised protocols for 24-hour urine collections, self-reported dietary assessment, measurement of cognitive performance and BP, combined with a structured statistical approach to model testing and adjustment, enhance the robustness of the results. However, this is an ageing cohort recruited from the Chianti region of Italy, and generalisability to other settings needs to be established. The cross-sectional design is an important limitation of our study, as causality of the associations cannot be determined. Some methodological issues to be considered in interpreting our findings also include potential measurement error in the self-reported dietary data. Additionally, we cannot rule out the effects of residual confounding on the analyses. It is important to consider the limitations surrounding the cognitive data used in this study. Although global cognition was measured via MMSE, frontal lobe function evaluation was limited to TMT-A and TMT-B due to data availability. It cannot be excluded that subtle deficits might have been detected with more extensive neuropsychological testing (Vinciguerra et al. 2020). It is important to note the limitations surrounding collection of 24-hour urinary samples at one time-point. Finally, the contribution of drinking water to the total dietary nitrate intake was not available in our database. However, nitrate intake from water accounts for about 5–15% of total nitrate intake (Ward et al. 2018) and there is no indication of a systematic difference in water intake between subjects; hence, the lack of adjustment for nitrate in drinking water is unlikely to modify our results.

## Conclusions

Urinary nitrate excretion was associated with reduced BP among the InCHIANTI study population and the results were independent of dietary sodium intake. However, this result requires further confirmation in longitudinal studies and carefully controlled physiological investigations. Similarly, the lack of association of dietary sodium and nitrate intake with cognitive function and dementia needs further testing in longitudinal analysis with prolonged follow-up time to be able to detect diet-driven changes in cognitive trajectories. The results confirm the potential role of nitrate as an important cardiovascular protective component of the diet, which may account for some of the cardiovascular benefits associated with adherence to healthy dietary patterns such as the Mediterranean Diet or Dietary Approach to Stop Hypertension (DASH diet).

## Supplementary Material

SM

Supplemental data for this article can be accessed online at https://doi.org/10.1080/09637486.2021.1993157.

## Figures and Tables

**Table 1.** Characteristics of InCHIANTI study participants aged >50 years at baseline (*n* = 989).

*N*	Mean (SD) or *n* (%)
Age, years	73.5 (8.8)
Sex, men	452 (45.7)
Years of School *(n* = 987) Highest educational level completed	5.6 (3.5)
Nothing	273 (27.6)
Elementary	513 (51.9)
Secondary	77 (7.8)
High school	50 (5.1)
Professional school	36 (3.6)
University or equivalent	27 (2.7)
Undocumented educational level Smoking status	13 (1.3)
Never smoked cigarettes	510 (57.4)
Former cigarette smoker	234 (26.4)
Current (within 3 years of interview) Diabetes	144 (16.2)
Definite	113 (11.4)
Possible Congestive heart failure	11 (1.1)
Definite	46 (4.7)
Possible Stroke	165 (16.7)
Definite	50 (5.1)
Possible	3 (0.3)
TIA Parkinson’s disease	14 (1.4)
Definite	13 (1.3)
Possible Myocardial infarction	9 (0.9)
Definite	36 (3.6)
Possible	4 (0.4)
Cancer Number of co-morbidities	50 (5.1)
(MI, CHF, stroke, cancer, PD, diabetes) 0	605 (61.2)
1–2	365 (36.9)
3–4	19 (1.9)
Systolic blood pressure, mmHg	145.2 (21.2)
Diastolic blood pressure, mmHg	82.7 (9.1)
Weight, kg	69.9 (12.9)
BMI, kg/m2 *(n* = 982)	27.2 (4.1)
eGFR, mL/min	67.6 (20.7)
CESD Depression Score (*n* = 884) (0–60) Physical activity (*n* = 861)	12.8 (8.9)
Low	160 (16.2)
Medium	410 (41.5)
High	356 (36.0)
Energy intake, kcal/day	1996.4 (602.2)
Sodium, mg/day	2357.9 (848.9)
Nitrate, mg/day (i = 973)	89.0 (73.3)
Urinary sodium, μmol/24 hr (*n* = 913)	139.5 (65.7)
Urinary nitrate μmol/24 hr (*n* = 895) Cognitive function	862.5 (734.7)
MMSE score (*n* = 989)	24.5 (5.2)
TMT A score (*n* = 889)	29.9 (16.5)
TMT B score (*n* = 721)	28.9 (18.2)
TMT A–B (*n* = 721)	1.72 (24.1)

Values expressed as estimated mean and standard deviation (SD) or frequency (*n*) and percentage (%). Where measurements were not obtained in the full set of 989 participants, the exact number of participants for the variable is stated in brackets under the variable name. *Key*: BMI: body mass Index; CESD: Centre for Epidemiological Studies Depression; eGFR: estimated glomerular filtration rate; MMSE: Mini Mental State Examination; TIA: transient ischaemic attack; TMT A: Trail Making Test A; TMT B: Trail Making Test B; TMT A–B: Difference between TMT A and TMT B test scores.

**Table 2.** Multiple linear regression to investigate the association between urinary and dietary measures of sodium and nitrate intake with cognitive performance.

	MMSE	TMT A	TMT B
Urinary Sodium (μmol/24 hr)			
Model 1	0.003 ± 0.002	−0.005 ± 0.009	−0.006 ± 0.011
	*p* = 0.120	*p* = 0.566	*p* = 0.554
Model 2	0.001 ± 0.001	−0.007 ± −0.009	−0.009 ± 0.011
	*p* = 0.537	*p* = 0.476	*p* = 0.406
Dietary Sodium Intake (mg/day)			
Model 1	<0.0001 ± 0.001	0.001 ± 0.001	<0.0001 ± 0.001
	*p* = 0.539	*p* = 0.304	*p* = 0.878
Model 2	<0.0001 ± 0.001	<0.001 ± 0.001	<0.0001 ± 0.001
	*p* = 0.212	*p* = 0.722	*p* = 0.988
Urinary Nitrate (μmol/24 hr)			
Model 1	<0.0001 ± 0.001	0.001 ± 0.001	−0.001 ± 0.001
	*p* = 0.750	*p* = 0.503	*p* = 0.508
Model 2	<0.0001 ± 0.001	<0.0001 ± 0.001	−0.001 ± 0.001
	*p* = 0.986	*p* = 0.925	*p* = 0.208
Dietary Nitrate Intake (mg/day)			
Model 1	0.001 ± 0.001	−0.002 ± 0.008	−0.006 ± 0.009
	*p* = 0.608	*p* = 0.782	*p* = 0.488
Model 2	0.001 ± 0.001	−0.004 ± 0.008	−0.006 ± 0.009
	*p* = 0.415	*p* = 0.693	*p* = 0.513

Associations were explored via multiple linear regression. Results expressed as estimate and standard error.

* *p* Value <0.05. Models were unadjusted (model 1) and adjusted for age, sex, disease count score (stroke, CHF, MI, PD, cancer and diabetes), medication use (drugs for acid-related disorders; diuretics for CHF or hypertension; ACE inhibitors (alone and in combination with diuretic); organic nitrates; aldosterone antagonists (diuretics); glucocorticoids for systemic use; antiepileptic; anti-Parkinson drugs; psycholeptics: typical antipsychotics; psycholeptics: atypical antipsychotics; psycholeptics: anxiolytics; psychoanaleptics: antidepressants; drugs for dementia), BMI, Physical Activity, Total Energy Intake, Smoking, Education, Depression, Kidney function, Blood pressure (SBP and DBP) (model 2).

*Key*: ACE: angiotensin-converting-enzyme; BMI: body mass index; CHF: chronic heart failure; DBP: diastolic blood pressure; MI: myocardial infarction; MMSE: Mini Mental State Examination; PD: Parkinson’s disease; TMT A: Trail Making Test A; TMT B: Trail Making Test B; SBP: systolic blood pressure.

**Table 3.** Multiple linear regression of the association sodium and nitrate intake with blood pressure (BP).

	SBP	DBP
Urinary sodium (μmol/24 hr)		
Model 1	−0.008 ± 0.011	−0.005 ± 0.005
	*p* = 0.542	*p* = 0.329
Model 2	−0.010 ± 0.011	−0.005 ± 0.005
	*p* = 0.371	*p* = 0.262
Dietary sodium intake (mg/day)		
Model 1	− <0.001 ± 0.001	<0.0001 ± 0.001
	*p* = 0.631	*p* = 0.968
Model 2	−0.001 ± 0.001	−0.0001 ± 0.001
	*p* = 0.638	*p* = 0.894
Urinary nitrate (μmol/24 hr)		
Model 1	−0.005 ± 0.001	−0.001 ± 0.001
	*p*= <0.001	*p* = 0.002
Model 2	−0.005 ± 0.001	−0.001 ± 0.001
	*p*= <0.001	*p* = 0.002
Dietary nitrate intake (mg/day)		
Model 1	−0.003 ± 0.010	0.005 ± 0.004
	*p* = 0.741	*p* = 0.188
Model 2	−0.004 ± 0.010	0.005 ± 0.004
	*p* = 0.678	*p* = 0.214

Associations were explored via multiple linear regression. Results expressed as estimate and standard error. Models were unadjusted (model 1) and adjusted for age, sex, disease count score (stroke, CHF, MI, PD, cancer and diabetes), BMI, Physical Activity, Total Energy Intake, Smoking, Kidney function, medication use (drugs for acid-related disorders; diuretics for CHF or hypertension; ACE inhibitors (alone and in combination with diuretic); organic nitrates; aldosterone antagonists (diuretics); glucocorticoids for systemic use) (model 2).

*Key*: BMI: body mass index; CHF: chronic heart failure; DBP: diastolic blood pressure; MI: myocardial infarction; PD: Parkinson’s disease; SBP: systolic blood pressure

**Table 4.** Logistic regression of the association between sodium and nitrate intake and risk of high blood pressure.

	SBP	DBP
Urinary sodium (μmol/24 hr) Model 1		
Low (n = 279)	1.00	1.00
(0–90.3)	(Reference)	(Reference)
Medium *(n* = 313)	1.223 (0.87–1.73)	0.919 (0.65–1.29)
(90.3–131.6)	*p* = 0.252	*p* = 0.627
High (*n* = 321)	0.981 (0.70–1.37)	0.885 (0.63–1.24)
(131.6 and above)	*p* = 0.911 Model 2	*p* = 0.481
Low (*n* = 279)	1.00	1.00
(0–90.3)	(Reference)	(Reference)
Medium (*n* = 313)	1.15 (0.80–1.66)	0.90 (0.63–1.31)
(90.3–131.6)	*p* = 0.458	*p* = 0.597
High (*n* = 321)	0.93 (0.67–1.34)	0.89 (0.62–1.28)
(131.6 and above)	*p* = 0.721	*p* = 0.540
Dietary sodium Intake (mg/day) Model 1		
Low (*n* = 309)	1.00	1.00
(0–1901.4)	(Reference)	(Reference)
Medium (*n* = 346)	1.286 (0.93–1.78)	1.076 (0.78–1.49)
(1901.4–2600.5)	*p* = 0.132	*p* = 0.659
High (*n* = 334)	1.095 (0.78–1.55)	0.904 (0.65–1.26)
(2600.5 and above)	*p* = 0.607 Model 2	*p* = 0.552
Low (*n* = 309)	1.00	1.00
(0–1901.4)	(Reference)	(Reference)
Medium (*n* = 346)	1.35 (0.93–1.97)	1.01 (0.70–1.46)
(1901.4–2600.5)	*p* = 0.115	*p* = 0.958
High (*n* = 334)	1.14 (0.72–1.82)	0.84 (0.53–1.35)
(2600.5 and above)	*p* = 0.563	*p* = 0.482
Urinary nitrate (μmol/24 hr) Model 1		
Low (*n* = 303)	1.00	1.00
(0–502.3)	(Reference)	(Reference)
Medium (*n* = 298)	0.656 (0.46–0.94)	0.795 (0.58–1.10)
(502.4–894.8)	*p* = 0.021	*p* = 0.166
High (*n* = 294)	0.416 (0.29–0.59)	0.769 (0.56–1.06)
(894.9 and above)	*p*= <0.0001 Model 2	*p* = 0.113
Low (*n* = 303)	1.00	1.00
(0–502.3)	(Reference)	(Reference)
Medium (*n* = 298)	0.57 (0.37–0.88)	0.75 (0.52–1.10)
(502.4–894.8)	*p* = 0.011	*p* = 0.140
High (*n* = 294)	0.31 (0.21–0.48)	0.68 (0.47–1.01)
(894.9 and above)	*p*= <0.0001	*p* = 0.054
Dietary nitrate intake (mg/day) Model 1		
Low (*n* = 319)	1.00	1.00
(0.00–502.3)	(Reference)	(Reference)
Medium (*n* = 325)	1.028 (0.73–1.43)	1.057 (0.76–1.47)
(502.4–894.8)	*p* = 0.869	*p* = 0.739
High (*n* = 329)	0.951 (0.69–1.32)	0.996 (0.72–1.38)
(894.9 and above)	*p* = 0.761 Model 2	*p* = 0.981
Low (*n* = 319)	1.00	1.00
(0.00–502.3)	(Reference)	(Reference)
Medium (*n* = 325)	1.07 (0.76–1.52)	1.11 (0.79–1.57)
(502.4–894.8)	*p* = 0.694	*p* = 0.549
High (*n* = 329)	1.00 (0.71–1.42)	0.97 (0.69–1.38)
(894.9 and above)	*p* = 0.979	*p* = 0.886

Associations were explored via logistic regression.

* Significantly (*p* < 0.05) higher risk of hypertension compared with the lowest tertile of urinary sodium/dietary sodium/urinary nitrate/dietary nitrate. Hypertension classified using European Society of Cardiology ESC guidelines – normal SBP < =139mmHg: Hypertension > =140mmHg; Normal DBP < =89mmHg: Hypertension > =90mmHg). Models were unadjusted (model 1) and adjusted for age, sex, disease count score (stroke, CHF, MI, PD, cancer and diabetes), BMI, Physical Activity, Total Energy Intake, Smoking, Kidney function, medication use (drugs for acid-related disorders; diuretics for CHF or hypertension; ACE inhibitors (alone and in combination with diuretic); organic nitrates; aldosterone antagonists (diuretics); glucocorticoids for systemic use) (model 2). *Key*: BMI: body mass index; CHF: chronic heart failure; DBP: diastolic blood pressure; MI: myocardial infarction; PD: Parkinson’s disease; SBP: systolic blood pressure.

**Table 5.** Logistic regression of the association between sodium and nitrate intake groups and risk of high blood pressure.

	SBP	DBP
Model 1		
Groups 4: LNHS	1.00	1.00
(*n* = 207)	(Reference)	(Reference)
Group 1: HNLS	0.49 (0.32–0.75)	0.95 (0.63–1.43)
(n = 205)	*p* = 0.001*	*p* = 0.806
Group 2: HNHS	0.48 (0.32–0.78)	0.93 (0.62–1.40)
(*n* = 205)	*p* = 0.001*	*p* = 0.725
Group 3: LNLS	0.83 (0.53–1.29)	1.15 (0.77–1.73)
(*n* = 205)	*p* = 0.829	*p* = 0.489
Model 2		
Groups 4: LNHS	1.00	1.00
(*n* = 207)	(Reference)	(Reference)
Group 1: HNLS	0.49 (0.32–0.76)	1.05 (0.68–1.64)
(*n* = 205)	*p* = 0.001*	*p* = 0.814
Group 2: HNHS	0.49 (0.32–0.77)	0.94 (0.61–1.46)
(*n* = 205)	*p* = 0.002*	*p* = 0.782
Group 3: LNLS	0.85 (0.54–1.35)	1.18 (0.77–1.82)
(*n* = 205)	*p* = 0.504	*p* = 0.435

Associations were explored via logistic regression.

* Significantly (*p* < 0.05) higher risk of hypertension compared with those in groups 2, 3 and 4 (reference). Hypertension classified using European Society of Cardiology ESC guidelines – Normal SBP < =139mmHg: Hypertension > =140mmHg; Normal DBP < =89mmHg: Hypertension > =90mmHg)

Models were unadjusted (model 1) and adjusted for age, sex, disease count score (stroke, CHF, MI, PD, cancer and diabetes), BMI, Physical Activity, Total energy Intake, Smoking, Kidney function and medication use (drugs for acid-related disorders; diuretics for CHF or hypertension; ACE inhibitors (alone and in combination with diuretic); organic nitrates; aldosterone antagonists (diuretics); glucocorticoids for systemic use) (model 2).

*Key*: BMI: body mass index; CHF: chronic heart failure; DBP: diastolic blood pressure; HNLS: high nitrate low sodium; HNHS: high nitrate high sodium; LNLS: low nitrate low sodium; LNHS: low nitrate high sodium; MI: myocardial infarction; PD: Parkinson’s disease; SBP: systolic blood pressure.

## Data Availability

The authors confirm that the data supporting the findings of this study are available within the article [and/or] its supplementary materials. The datasets used and/or analysed during the cur-rent study are available from the corresponding author on reasonable request.
