# Supplementary material for: Independent and interactive associations of dietary nitrate and salt intake with blood pressure and cognitive function: a cross-sectional analysis in the InCHIANTI study
Source: Int J Food Sci Nutr. Author manuscript; Available in PMC 2025 Jun 12. (PMC12159797; doi:10.1080/09637486.2021.1993157)
Supplement: SM [file NIHMS2023637-supplement-SM.pdf]

Online Supplementary Material

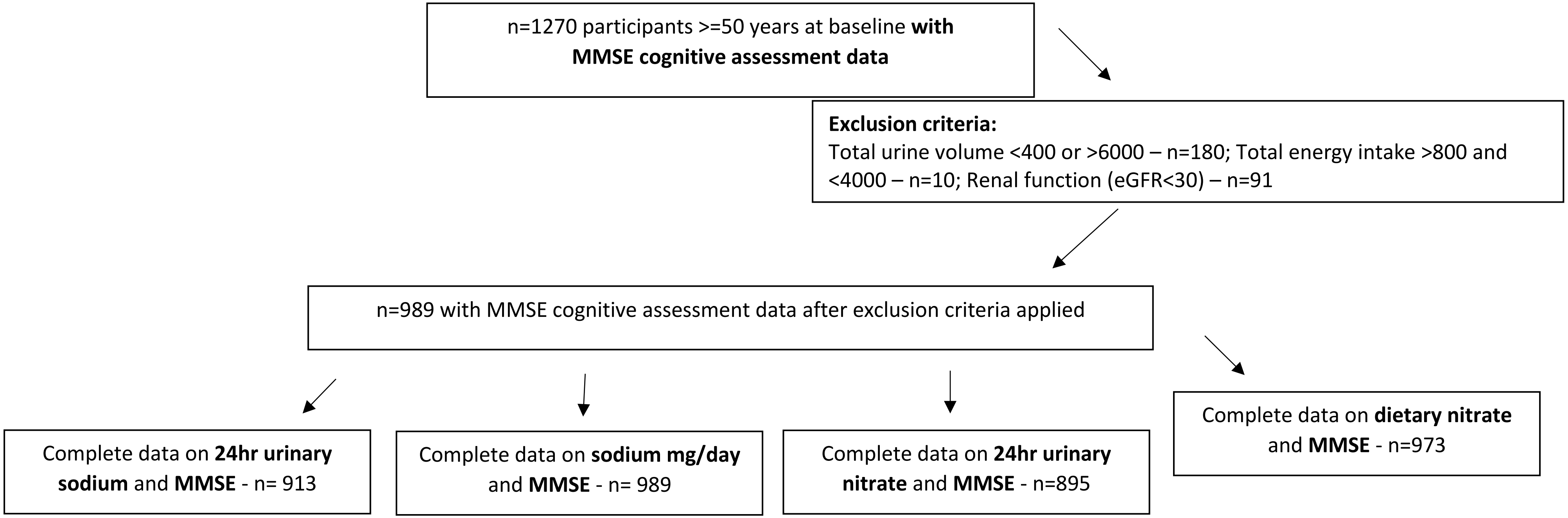

Figure 1a: Flowchart of study participants eligible for inclusion

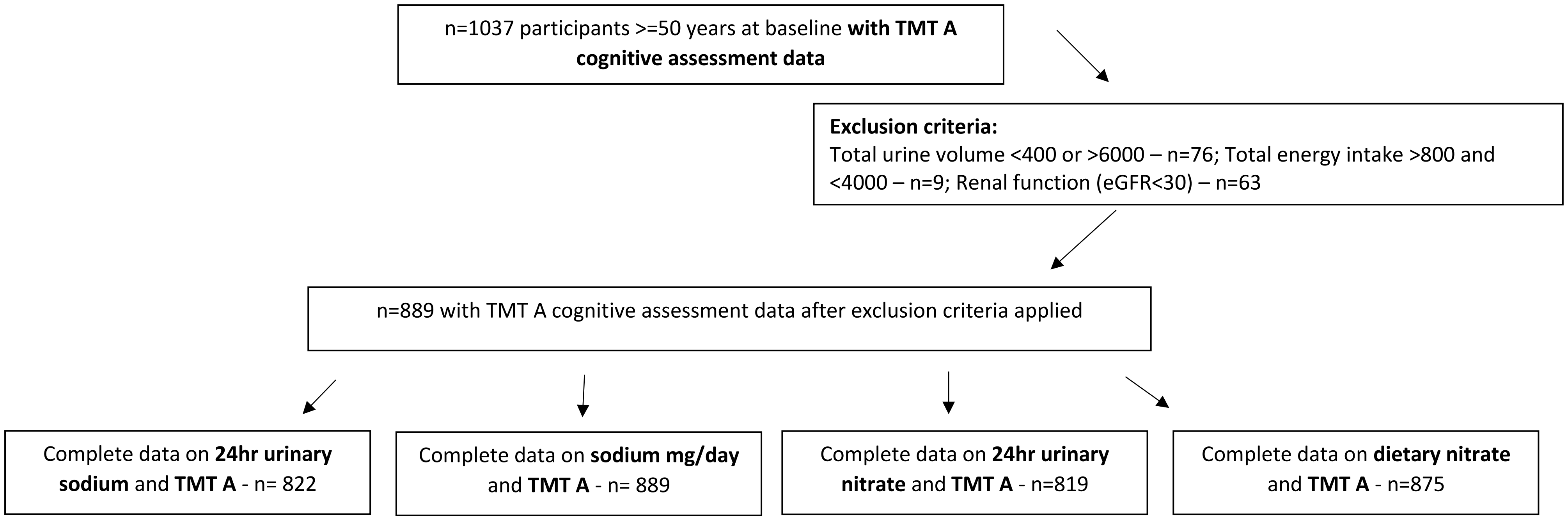

Figure 1b: Flowchart of study participants eligible for inclusion

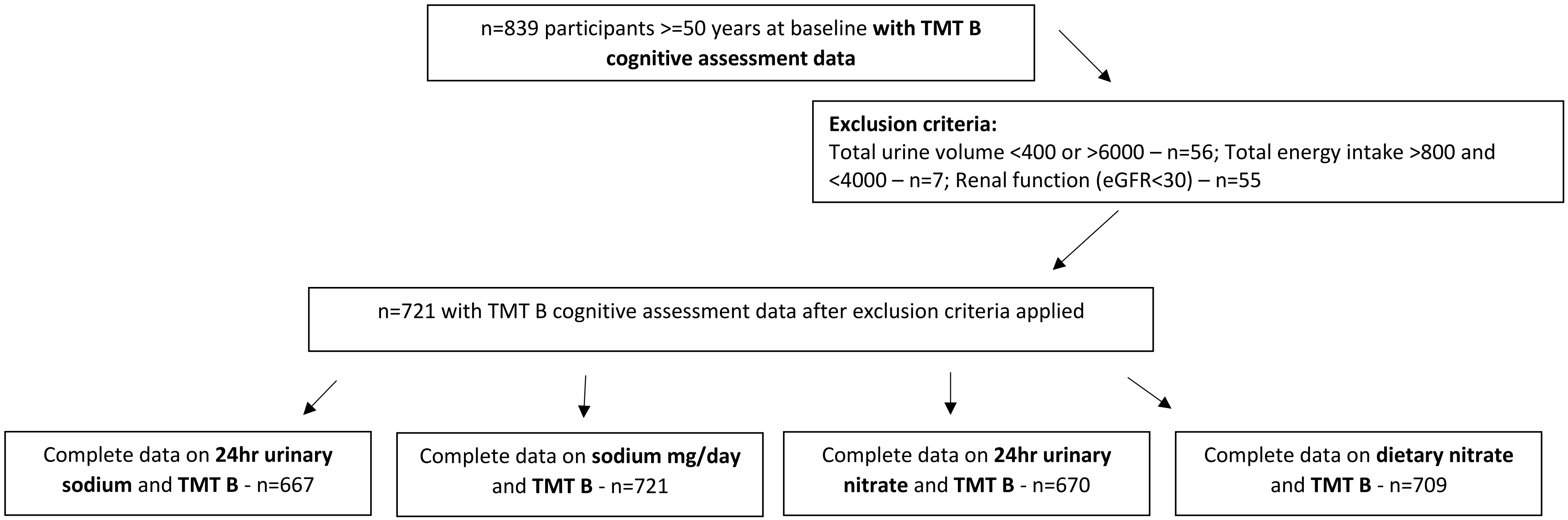

Figure 1c: Flowchart of study participants eligible for inclusion

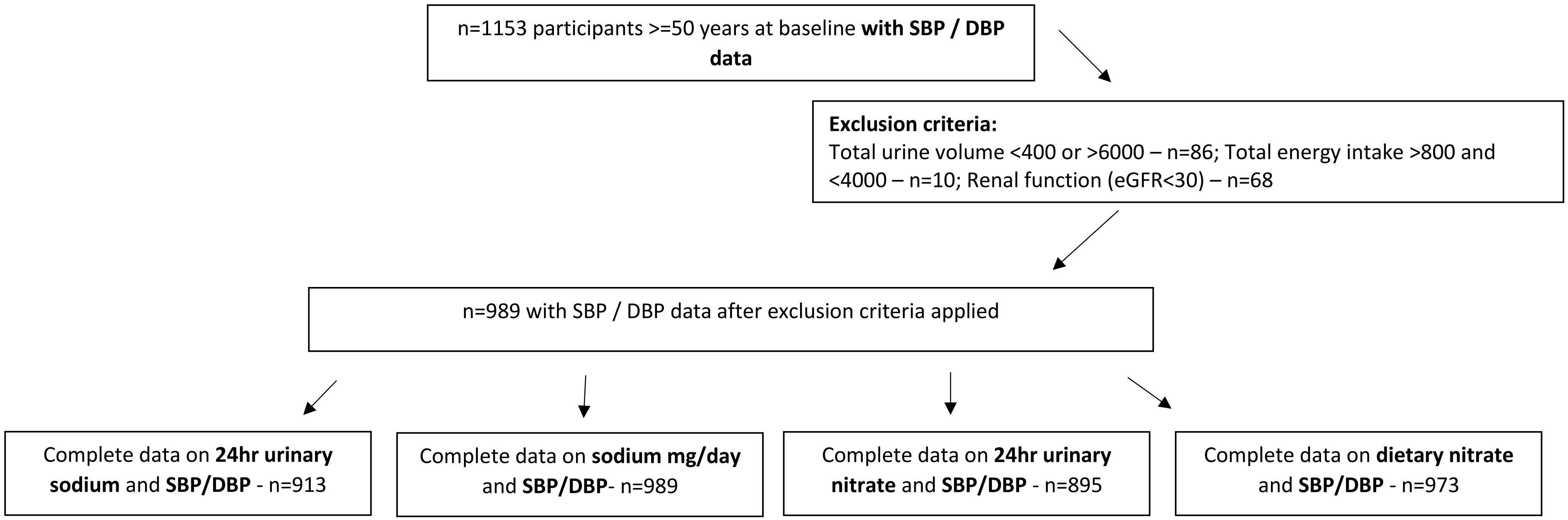

Figure 1d: Flowchart of study participants eligible for inclusion

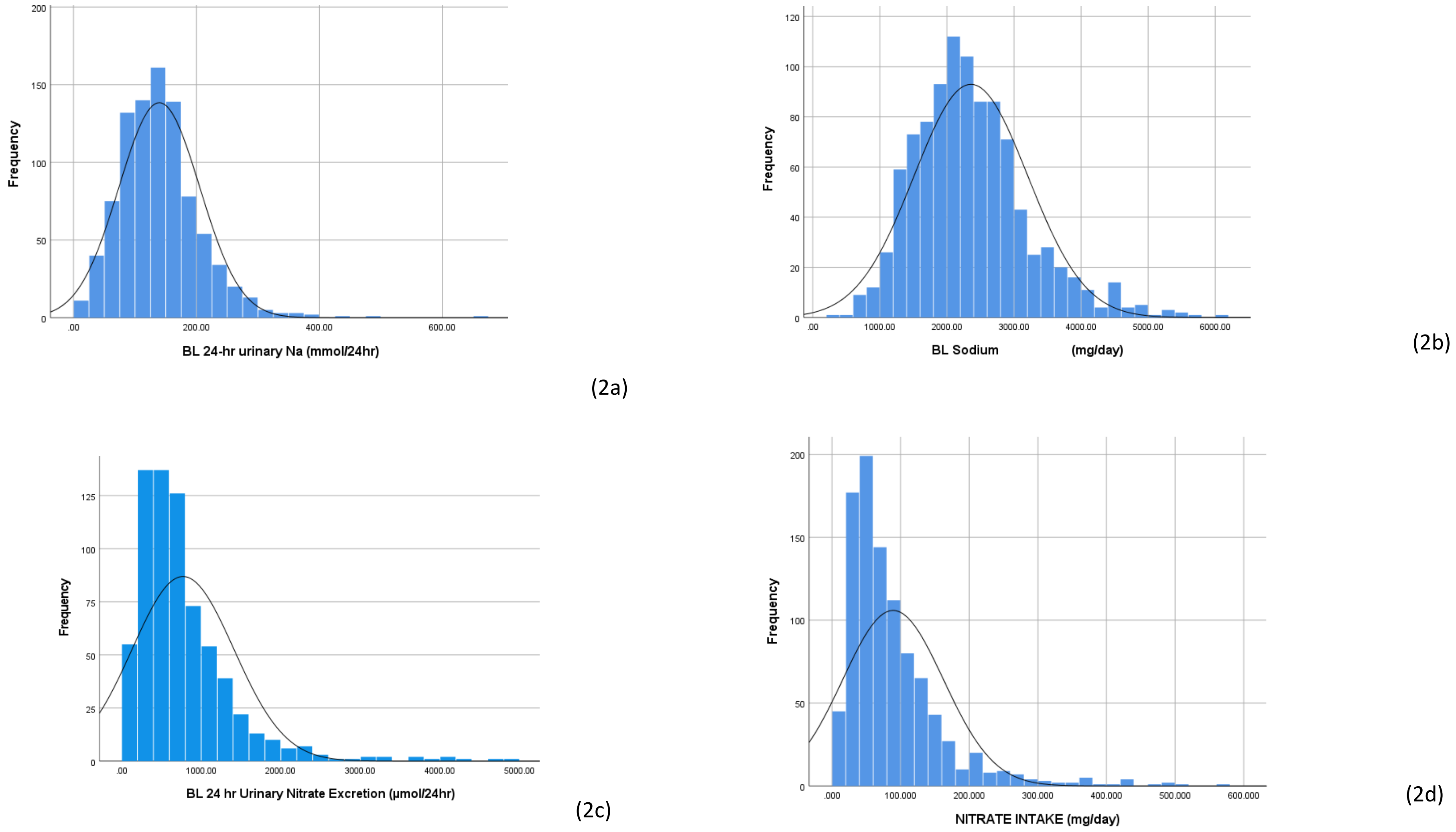

Figure 2 (a-d): Histograms of distribution of dietary and 24-hour urinary concentrations for nitrate and sodium data.

Table 1: Logistic regression of the association between sodium and nitrate intake and poor cognitive performance

	MMSE	TMT A	TMT B	TMT A-B
**Urinary Sodium (mmol/24hr)** **Model 1**				
**Low (n=279)** (0-90.3)	1.00 (reference)	1.00 (reference)	1.00 (reference)	1.00 (reference)
**Medium (n=313)** (90.3-131.6)	0.98 (0.70-1.39) *p*=0.920	1.22 (0.81-1.83) *p*=0.342	0.92 (0.59-1.43) *p*=0.709	0.61 (0.39-0.97) P=0.038
**High (n=321)** (131.6 and above)	0.66 (0.47-0.96) *p*=0.03	1.18 (0.79-1.77) *p*=0.426	0.74 (0.47-1.17) *p*=0.200	1.17 (0.79-1.76) *p*=0.426
**Model 2**				
**Low (n=279)** (0-90.3)	1.00 (reference)	1.00 (reference)	1.00 (reference)	1.00 (reference)
**Medium (n=313)** (90.3-131.6)	1.10 (0.68-1.76) *p*=0.703	1.18 (0.76-1.84) *p*=0.465	0.69 (0.42-1.15) *p*=0.096	0.55 (0.33-0.92) P=0.022
**High (n=321)** (131.6 and above)	0.79 (0.49-1.28) *p*=0.339	1.24 (0.80-1.91) *p*=0.334	0.76 (0.46-1.23) *p*=0.273	1.19 (0.76-1.87) *p*=0.449
**Sodium mg/day (based on frequency distribution)** **Model 1**				
**Low (n=309)** (0-1901.4)	1.00 (reference)	1.00 (reference)	1.00 (reference)	1.00 (reference)
**Medium (n=346)** (1901.4-2600.5)	0.93 (0.66-130) *p*=0.665	0.81 (0.55-1.18) *p*=0.805	0.76 (0.49-1.17) *p*=0.207	0.80 (0.53-1.20) *p*=0.284
**High (n=334)** (2600.5 and above)	0.95 (0.68-1.33) *p*=0.295	0.84 (0.57-1.23) *p*=0.371	0.91 (0.60-1.39) *p*=0.668	0.78 (0.52-1.17) *p*=0.238
**Model 2**				
**Low (n=309)** (0-1901.4)	1.00 (reference)	1.00 (reference)	1.00 (reference)	1.00 (reference)
**Medium (n=346)** (1901.4-2600.5)	0.76 (0.47-1.23) *p*=0.257	0.77 (0.50-1.20) *p*=0.245	0.74 (0.45-1.24) *p*=0.256	0.83 (0.51-1.34) *p*=0.441
**High (n=334)** (2600.5 and above)	0.67 (0.37-1.22) *p*=0.193	0.96 (0.55-1.66) *p*=0.890	1.07 (0.57-1.98) *p*=0.840	0.94 (0.51-1.71) *p*=0.839

Table 1: Logistic regression of the association between sodium and nitrate intake and poor cognitive performance

	MMSE	TMT A	TMT B	TMT A-B
**Urinary Nitrate μmol /24hr (based on frequency distribution)**				
**Model 1**				
**Low (n=303)**	1.00	1.00	1.00	1.00
(0-502.3)	(reference)	(reference)	(reference)	(reference)
**Medium (n=298)**	1.09 (0.77-1.56)	0.75 (0.51-1.10)	1.42 (0.91-2.22)	1.33 (0.85-2.10)
(502.4-894.8)	*p*=0.624	*p*=0.143	*p*=0.121	*p*=0.217
**High (n=294)**	1.31 (0.92-1.85)	0.62 (0.41-1.93)	1.39 (0.77-1.93)	1.55 (0.98-2.43)
(894.9 and above)	*p*=0.136	=0.109	*p*=0.396	*p*=0.06
**Model 2**				
**Low (n=303)**	1.00	1.00	1.00	1.00
(0-502.3)	(reference)	(reference)	(reference)	(reference)
**Medium (n=298)**	1.15 (0.71-2.11)	0.76 (0.50-1.16)	1.58 (0.97-2.58)	1.09 (0.67-1.78)
(502.4-894.8)	*p*=0.565	*p*=0.207	*p*=0.068	*p*=0.716
**High (n=294)**	1.29 (0.79-2.11)	0.73 (0.47-1.13)	1.30 (0.77-2.19)	1.21 (0.74-2.00)
(894.9 and above)	*p*=0.306	*p*=0.161	*p*=0.322	*p*=0.441
**Dietary Nitrate mg/day (based on frequency distribution)**				
**Model 1**				
**Low (n=319)**	1.00	1.00	1.00	1.00
(0.00-502.3)	(reference)	(reference)	(reference)	(reference)
**Medium (n=325)**	1.08 (0.77-1.51)	1.30 (0.89-1.92)	1.05 (0.68-1.63)	1.48 (0.96-2.27)
(502.4-894.8)	*p*=0.657	*p*=0.178	*p*=0.814	P=0.069
**High (n=329)**	1.00 (0.71-1.40)	1.11 (0.75-1.64)	1.06 (0.69-1.64)	1.32 (0.86-2.02)
(894.9 and above)	*p*=0.996	*p*=0.609	*p*=0.781	*p*=0.211
**Model 2**				
**Low (n=319)**	1.00	1.00	1.00	1.00
(0.00-502.3)	(reference)	(reference)	(reference)	(reference)
**Medium (n=325)**	0.94 (0.60-1.48)	1.35 (0.89-2.04)	1.11 (0.69-1.79)	1.48 (0.94-2.35)
(502.4-894.8)	*p*=0.800	*p*=0.149	*p*=0.665	P=0.090
**High (n=329)**	0.85 (0.54-1.33)	1.12 (0.74-1.71)	1.14 (0.70-1.83)	1.21 (0.70-1.79)
(894.9 and above)	*p*=0.475	*p*=0.586	*p*=0.602	*p*=0.635

Associations were explored via logistic regression. ∗Significantly ($P < 0.05$) higher risk of poor cognitive performance compared with the lowest tertile of urinary sodium / dietary sodium / urinary nitrate / dietary nitrate. MMSE - Poor performance was defined as a score in the bottom 20th percentile of the population distribution for MMSE scores as lower scores are indicative of poorer performance. TMT A and TMT B - Poor performance was defined as a score in the 20th percentile of the population distribution as higher scores demonstrate poorer performance (more time taken to complete). Models were unadjusted (model 1) and adjusted for age, sex, disease count score (stroke, CHF, MI, PD, cancer and diabetes), medication use (drugs for acid-related disorders; diuretics for CHF or hypertension; ACE inhibitors (alone and in combination with diuretic); organic nitrates; aldosterone antagonists (diuretics); glucocorticoids for systemic use; antiepileptic; anti-Parkinson drugs; psycholeptics: typical antipsychotics; psycholeptics: atypical antipsychotics; psycholeptics: anxiolytics; psychoanaleptics: antidepressants; drugs for dementia), BMI, Physical Activity, Total energy Intake, Smoking, Education, Depression, Kidney function, Blood pressure (SBP and DBP) (model 2).
Key: **BMI** Body mass Index; **CHF** Chronic Heart Failure; **DBP** Diastolic Blood Pressure; **MI** Myocardial infarction; **MMSE** Mini Mental State Examination; **PD** Parkinson's Disease; **TMT A** Trail Making Test A; **TMT B** Trail Making Test B; **SBP** Systolic Blood Pressure

Table 2: Binary logistic regression of the association between sodium and nitrate intake groups (urinary concentrations) and poor cognitive performance

	MMSE	TMT A	TMT B	TMT A-B
**Model 1**				
**Group 4: LNHS (n=207)**	1.00 (reference)	1.00 (reference)	1.00 (reference)	1.00 (reference)
**Group 1: HNLS (n=205)**	1.72 (1.13-2.65) *p*=0.012	0.49 (0.30-1.80) *p*=0.100	1.05 (0.61-1.80) *p*=0.858	1.91 (0.72-1.97) *p*=0.494
**Group 2: HNHS (n=205)**	1.24 (0.78-1.91) *p*=0.342	0.60 (0.35-1.91) *p*=0.200	0.97 (0.57-1.68) *p*=0.922	1.05 (0.63-1.75) *p*=0.861
**Group 3: LNLS (n=205)**	1.36 (0.88-2.09) *p*=0.167	0.80 (0.51-1.25) *p*=0.330	1.01 (0.59-1.74) *p*=0.967	0.68 (0.39-1.19) *p*=0.177
**Model 2**				
**Group 4: LNHS (n=207)**	1.00 (reference)	1.00 (reference)	1.00 (reference)	1.00 (reference)
**Group 1: HNLS (n=205)**	1.46 (0.83-2.59) p=0.192	0.48 (0.28-1.18) *p*=0.119	1.09 (0.60-1.99) *p*=0.767	0.97 (0.55-1.71) *p*=0.915
**Group 2: HNHS (n=205)**	0.84 (0.46-1.50) P=0.547	0.68 (0.41-1.12) *p*=0.132	0.86 (0.47-1.59) *p*=0.639	0.90 (0.51-1.58 *p*=0.717
**Group 3: LNLS (n=205)**	1.12 (0.64-1.97) P=0.698	0.85 (0.52-1.36) *p*=0.491	0.97 (0.53-1.75) *p*=0.908	0.64 (0.35-1.17) *p*=0.147

Associations were explored via logistic regression. *Significantly ($P < 0.05$) higher risk of poor cognitive performance compared with the rest of the study population (reference). MMSE - Poor performance was defined as a score in the bottom 20th percentile of the population distribution for MMSE scores as lower scores are indicative of poorer performance. TMT A and TMT B - Poor performance was defined as a score in the 20th percentile of the population distribution as higher scores demonstrate poorer performance (more time taken to complete).

Model 1 – unadjusted; Model 2 –adjusted for age, sex, disease count score (stroke, CHF, MI, PD, cancer and diabetes), medication use (drugs for acid-related disorders; diuretics for CHF or hypertension; ACE inhibitors (alone and in combination with diuretic); organic nitrates; aldosterone antagonists (diuretics); glucocorticoids for systemic use; antiepileptic; anti-Parkinson drugs; psycholeptics: typical antipsychotics; psycholeptics: atypical antipsychotics; psycholeptics: anxiolytics; psychoanaleptics: antidepressants; drugs for dementia), BMI, Physical Activity, Total energy Intake, Smoking, Education, Depression, Kidney function, Blood pressure (SBP and DBP).

Key: **BMI** Body mass Index; **CHF** Chronic Heart Failure; **DBP** Diastolic Blood Pressure; **MI** Myocardial infarction; **MMSE** Mini Mental State Examination; **PD** Parkinson's Disease; **TMT A** Trail Making Test A; **TMT B** Trail Making Test B; **SBP** Systolic Blood Pressure; **HNLS** High Nitrate and Low Sodium; **HNHS** High Nitrate and High Sodium; **LNLS** Low Nitrate and Low Sodium; **LNHS** Low Nitrate and High Sodium

Table 3: Binary logistic regression of the association between sodium and nitrate intake groups (FFQ data) and poor cognitive performance

	MMSE	TMT A	TMT B	TMT A-B
**Model 1**				
**Group 4: LNHS (n=207)**	1.00 (reference)	1.00 (reference)	1.00 (reference)	1.00 (reference)
**Group 1: HNLS (n=205)**	1.03 (0.70-1.53) *p*=0.865	1.22 (0.78-1.90) *p*=0.379	1.21 (0.73-1.99) *p*=0.456	1.30 (0.81-2.10) *p*=0.284
**Group 2: HNHS (n=205)**	1.09 (0.74-1.59) *p*=0.676	1.03 (0.66-1.61) *p*=0.906	1.27 (0.78-2.07) *p*=0.345	1.05 (0.65-1.71) *p*=0.842
**Group 3: LNLS (n=205)**	1.14 (0.77-1.68) *p*=0.525	1.25 (0.80-1.95) *p*=0.328	1.00 (0.60-1.69) *p*=0.992	1.25 (0.76-2.03) *p*=0.367
**Model 2**				
**Group 4: LNHS (n=207)**	1.00 (reference)	1.00 (reference)	1.00 (reference)	1.00 (reference)
**Group 1: HNLS (n=205)**	1.05 (0.67-1.65) *p*=0.819	1.18 (0.74-1.89) *p*=0.479	1.01 (0.54-1.91) *p*=0.972	1.41 (0.69-1.89) *p*=0.609
**Group 2: HNHS (n=205)**	1.17 (0.76-1.81) *p*=0.676	1.00 (0.63-1.60) *p*=0.987	1.32 (0.76-2.27) *p*=0.325	0.96 (0.57-1.60) *p*=0.875
**Group 3: LNLS (n=205)**	1.33 (0.86-2.06) *p*=0.202	1.23 (0.77-1.95) *p*=0.383	0.88 (0.47-1.67) *p*=0.711	1.16 (0.70-1.92) *p*=0.568

Associations were explored via logistic regression. ∗Significantly ($P < 0.05$) higher risk of poor cognitive performance compared with the rest of the study population (reference). MMSE - Poor performance was defined as a score in the bottom 20th percentile of the population distribution for MMSE scores as lower scores are indicative of poorer performance. TMT A and TMT B - Poor performance was defined as a score in the 20th percentile of the population distribution as higher scores demonstrate poorer performance (more time taken to complete). Model 1 – unadjusted; Model 2 –adjusted for age, sex, disease count score (stroke, CHF, MI, PD, cancer and diabetes), medication use (drugs for acid-related disorders; diuretics for CHF or hypertension; ACE inhibitors (alone and in combination with diuretic); organic nitrates; aldosterone antagonists (diuretics); glucocorticoids for systemic use; antiepileptic; anti-Parkinson drugs; psycholeptics: typical antipsychotics; psycholeptics: atypical antipsychotics; psycholeptics: anxiolytics; psychoanaleptics: antidepressants; drugs for dementia), BMI, Physical Activity, Total energy Intake, Smoking, Education, Depression, Kidney function, Blood pressure (SBP and DBP).

Key: **BMI** Body mass Index; **CHF** Chronic Heart Failure; **DBP** Diastolic Blood Pressure; **MI** Myocardial infarction; **MMSE** Mini Mental State Examination; **PD** Parkinson's Disease; **TMT A** Trail Making Test A; **TMT B** Trail Making Test B; **SBP** Systolic Blood Pressure; **HNLS** High Nitrate and Low Sodium; **HNHS** High Nitrate and High Sodium; **LNLS** Low Nitrate and Low Sodium; **LNHS** Low Nitrate and High Sodium

Table 4: Logistic regression of the association between sodium and nitrate intake groups (FFQ data) and risk of high BP

	SBP	DBP
**Model 1**		
**Groups 4: LNHS (n=207)**	1.00 (reference)	1.00 (reference)
**Group 1: HNLS (n=205)**	0.93 (0.64-1.36) *p*=725	1.05 (0.72-1.53) *p*=0.800
**Group 2: HNHS (n=205)**	1.00 (0.69-1.46) *p*=0.988	0.96 (0.66-1.39) *p*=0.829
**Group 3: LNLS (n=205)**	0.93 (0.64-1.37) *p*=0.724	0.94 (0.64-1.37) *p*=0.743
**Model 2**		
**Groups 4: LNHS (n=207)**	1.00 (reference)	1.00 (reference)
**Group 1: HNLS (n=205)**	0.96 (0.61-1.51) *p*=0.852	0.99 (0.63-1.56) *p*=0.973
**Group 2: HNHS (n=205)**	1.07 (0.72-1.59) *p*=0.727	0.94 (0.63-1.38) *p*=0.737
**Group 3: LNLS (n=205)**	0.93 (0.59-1.45) *p*=0.751	0.89 (0.57-1.41) *p*=0.635

Associations were explored via logistic regression. ∗Significantly (P < 0.05) higher risk of hypertension compared with those in groups 2, 3 and 4 (reference). Hypertension classified using European Society of Cardiology ESC guidelines - Normal SBP <=139mmHg: Hypertension >=140mmHg; Normal DBP <=89mmHg: Hypertension >=90mmHg)

Model 1 – unadjusted; Model 2 – adjusted for age, sex, disease count score (stroke, CHF, MI, PD, cancer and diabetes), BMI, Physical Activity, Total energy Intake, Smoking, Kidney function, medication use (drugs for acid-related disorders; diuretics for CHF or hypertension; ACE inhibitors (alone and in combination with diuretic); organic nitrates; aldosterone antagonists (diuretics); glucocorticoids for systemic use).

Key: HNLS High Nitrate Low Sodium; **HNHS**; High Nitrate High Sodium; **LNLS** Low Nitrate Low Sodium; **LNHS** Low Nitrate High Sodium.

Table 5: Interactive effects of differences in 24-hour urinary concentrations of nitrate and sodium on cognitive function and resting blood pressure. Analysis of covariance was conducted to evaluate differences between the groups.

	MMSE	TMT A	TMT B	SBP	DBP
**Model 1**					
**Between Groups**	$F(3,818)=0.873$ $p=0.454$	$F(3,750)=1.687$ $P=0.168$	$F(3,614)=1.805$ $p=0.145$	$F(3,818)=7.357$ $p=<0.001$*	$F(3,818)=2.835$ $p=0.037$*
**Model 2**					
**Between Groups**	$F(3,712)=2.177$ $p=0.089$	$F(3,651)=1.058$ $p=0.366$	$F(3,531)=1.516$ $p=0.209$	$F(3,744)=6.600$ $p=<0.001$*	$F(3,744)=2.761$ $p=0.021$*

Analyses were unadjusted (model 1) and adjusted for age, sex, disease count score (stroke, CHF, MI, PD, cancer and diabetes), BMI, Physical Activity, Total energy Intake, Smoking, Kidney function and medication use (drugs for acid-related disorders; diuretics for CHF or hypertension; ACE inhibitors (alone and in combination with diuretic); organic nitrates; aldosterone antagonists (diuretics); glucocorticoids for systemic use) (model 2). Groups were: HNLS High Nitrate Low Sodium; HNHS; High Nitrate High Sodium; LNLS Low Nitrate Low Sodium; LNHS Low Nitrate High Sodium.
